# Use of a Wearable Self-Tracking Instrument by Refugees With Complex Posttraumatic Stress Disorder: A Qualitative Study of Psychotherapeutic Mediation and Engagement

**DOI:** 10.2196/70511

**Published:** 2025-07-09

**Authors:** Lisa Groenberg Riisager, Stine Bjerrum Moeller, Jakob Eg Larsen, Thomas Blomseth Christiansen, Jesper Aagaard, Lotte Huniche

**Affiliations:** 1 Department of Psychology, Faculty of Health Sciences, University of Southern Denmark, Campusvej 55, Odense, 5230, Denmark, 45 65501000; 2 Department of Multidisciplinary Trauma Treatment, Mental Health Services in the Region of Southern Denmark, Middelfart, Denmark; 3 Department of Applied Mathematics and Computer Science Cognitive Systems, Technical University of Denmark, Kongens Lyngby, Denmark; 4 Konsulent Blomseth, Hjoerring, Denmark; 5 Department of Psychology and Behavioral Sciences, Aarhus University, Aarhus, Denmark

**Keywords:** wearable technology, self-tracking, One Button Tracker, psychotherapy, engagement, postphenomenology, complex PTSD, personalized treatment, refugees, complex posttraumatic stress disorder

## Abstract

**Background:**

Wearable self-tracking technologies are increasingly used in mental health care to enhance engagement and personalize treatment. However, most existing instruments focus on passive data collection or predefined symptom monitoring. Less attention has been given to tools that enable patients to actively track personally meaningful, self-defined mental health experiences as part of psychotherapy, particularly in vulnerable populations such as refugees with complex posttraumatic stress disorder (CPTSD).

**Objective:**

This study aimed to explore how the One Button Tracker (OBT), a novel single-purpose wearable self-tracking instrument that enables in-the-moment active registration of self-defined, personally relevant mental health phenomena, supports therapeutic engagement among refugees receiving psychotherapeutic treatment for CPTSD.

**Methods:**

This qualitative study was part of a larger participatory action research project conducted from 2022 to 2024 at a specialized clinic for trauma-affected refugees in Denmark. A total of 9 adult refugees diagnosed with CPTSD used the OBT during psychotherapy to actively track a personally relevant and collaboratively defined target phenomenon through a button press. The OBT provided vibrotactile feedback and stored timestamped data for therapeutic use. A total of 25 semistructured interviews were conducted across 3 time points: before, during, and after treatment. Reflexive thematic analysis guided by a postphenomenological framework was used to explore how the technologies mediate experience.

**Results:**

Participants (6 women and 3 men, median age 47 years, IQR 31–57 years) had lived in Denmark for an average of 21.4 years. The duration of OBT use ranged from 22 to 366 days. Participants tracked between 2 and 14 different phenomena and registered between 37 and 4733 events in total during their courses of treatment. Thematic analysis revealed five central themes that captured the multistable character of the OBT: (1) from external instrument to extension of the self, (2) mental switch, (3) faithful companion, (4) scarlet letter, and (5) emergency lifeline. Patients described the OBT as a meaningful anchor in distressing moments, enhancing emotional regulation, self-awareness, and continuity between sessions. The OBT’s vibrotactile feedback was experienced as affirming and responsive, reinforcing a sense of being acknowledged and connected, even in the absence of direct therapist contact. However, the visibility of the OBT also introduced challenges, including stigma and altered social dynamics.

**Conclusions:**

The OBT functioned as an active mediator in therapy. It supported in-the-moment tracking of personal experiences, encouraged agency and emotional regulation, and helped patients feel connected to their therapist outside of sessions. The vibrotactile feedback played a key role in how the OBT was embodied and interpreted. These findings highlight the value of designing digital mental health technologies incorporating active self-tracking that emphasize relational use, experiential meaning, and personalization. A focus on simplistic design, adaptability, and patient-defined use may enhance therapeutic relevance, especially in settings where stigma or complexity limits engagement.

## Introduction

### Background

Wearable self-tracking technologies are increasingly recognized as valuable tools in mental health care, offering innovative approaches to enhance patient engagement, promote empowerment, and personalize treatment [1,2]. The rapid advancement of digital health technologies, particularly smartphones and wearable devices, has eased the integration of these tools into daily life, providing users with accessible means to monitor their health and well-being [3].

These wearable technologies are grounded in the principles of ubiquitous computing [4] and often rely on passive data collection, such as heart rate, sleep patterns, body temperature, and physical activity [5]. This passive monitoring approach minimizes the burden on patients while generating clinically relevant data that can be used for diagnostic and clinical purposes [1,6,7].

Within the field of digital mental health, 2 distinct types of data collection are recognized, namely passive and active. Passive data collection involves the automatic gathering of information without requiring user intervention, thus facilitating continuous monitoring of health metrics. In contrast, active data collection necessitates intentional engagement from users, allowing them to log specific experiences and emotions in real time, known as Ecological Momentary Assessment. This can be achieved through techniques such as electronic diaries [8] or the use of smartphone apps that prompt users to engage in assessments and interventions, known as Ecological Momentary Assessment and Intervention [9].

However, despite the advantages of both passive and active data collection methods, there is a tendency in current research to prioritize data collection at the expense of exploring how wearable self-tracking technologies can actively mediate the therapeutic process (eg, [10-12]). While there is an increasing focus on delivering treatment in patients’ daily lives and natural settings [13], interventions often consist of predefined assessment points with limited personalization [9]. This narrow approach may lead to missed opportunities to personalize treatment and use digital health technologies for in-the-moment engagement during the treatment process.

Engaging patients in tracking their subjective experiences as they occur can redirect focus to the present, fostering self-awareness, emotional regulation, and agency while producing personalized, fine-grained data [14]. By facilitating engagement in therapeutic interventions, wearable self-tracking technologies can empower patients to access support precisely when they need it, thereby enhancing their ability to navigate challenges in their daily lives. This dual role, acting as both data collectors and active mediators in therapeutic interventions, remains underexplored in mental health care research. A better understanding of this could reveal how wearable self-tracking technologies, when thoughtfully integrated, can extend therapeutic support beyond the clinical setting into patients’ everyday lives, ensuring they can engage in personalized interventions at the moments that matter most.

### The One Button Tracker: A Novel Approach to Engagement

This study introduces the One Button Tracker (OBT), a research prototype of a single-purpose wearable self-tracking instrument specifically for in-the-moment registration of a self-chosen subjectively experienced mental health phenomenon [14]. In contrast to multifunctional devices, such as smartphones or smartwatches, the OBT has one specific purpose: tracking self-defined mental health phenomena, while reducing cognitive and technical barriers.

**Figure 1.**
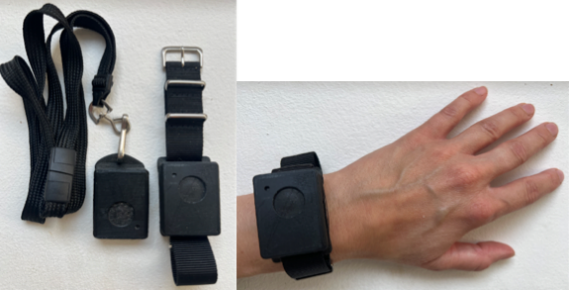
The One Button Tracker, a novel single-purpose wearable self-tracking instrument used in this study, shown configured with a neck strap and wristband.

The OBT is compact, measuring 41×31×12.5 mm, and operates with a user-friendly single-button interface (Figure 1). This design enables patients to record occurrences of their self-defined mental health phenomenon with a button press, promoting immediacy and precision in data collection. Each button press captures a timestamp and duration of the event [15] in the OBT’s internal storage [14]. These data can be accessed through a web-based data visualization tool collaboratively analyzed during therapy sessions.

Figure 1 displays the OBT that was used by refugee patients diagnosed with Complex Posttraumatic Stress Disorder during a clinical qualitative study conducted at the Clinic for Trauma and Torture Survivors in Denmark between 2022 and 2024. Emphasizing user adaptability, the OBT can be integrated into daily life without visual engagement, ensuring minimal disruption to routines. The instrument can be carried in a pocket, worn around the neck, or strapped to the wrist, catering to individual preferences (Figure 1). Furthermore, strong vibrotactile feedback accompanies each button press, providing tangible interaction with the instrument. This unique feature enhances the tracking experience, promoting user engagement without the need for visual attention.

### Addressing an Epistemic Gap in Mental Health Technology

Despite a growing interest in wearable technologies in mental health care [1], a significant epistemic gap remains: how do these tools mediate therapeutic experiences and shape patients’ perceptions of themselves and their mental health? Traditionally, wearable technology research has focused on measuring behavioral data, neglecting the dynamic, lived interactions between patients and these tools. In line with Torous et al [16] recommendations to define engagement in digital health in terms of the process rather than through behavioral measures, this study focuses on the OBT as an active mediator in the therapeutic process. By exploring the dynamic interactions between patients and the OBT, we aim to uncover how the instrument supports engagement, self-awareness, and becomes part of the therapeutic process. To guide this exploration, we turn to postphenomenology, a philosophy of technology developed by Ihde [17] and expanded by Verbeek [18] to provide us with a theoretical framework for understanding how technologies mediate human experiences.

### Research Question

The research question “How does the OBT mediate the psychotherapeutic process in everyday life?” was asked. Specifically, we explore how patients engage with the OBT during their daily lives, what the OBT means to them, and how it enables or hinders recovery in their treatment process.

## Methods

### Participants

This study was part of a larger participatory action research project aimed at co-developing a self-tracking assisted psychotherapy treatment concept for refugees diagnosed with complex posttraumatic stress disorder (CPTSD) [14]. The study was conducted at the Clinic for Trauma and Torture Survivors (CTTS) in the Region of Southern Denmark, a specialized outpatient facility within the Danish public health care system that provides interdisciplinary mental health care to approximately 550 trauma-affected refugees annually. The clinic offers evidence-based treatment, including psychotherapy and psychiatric support, for refugees with trauma-related mental health conditions.

Recruitment was conducted through routine clinical pathways. Psychologists at the clinic introduced the study to eligible patients either during the intake process or in the context of ongoing treatment. Some patients were already engaged in therapy for CPTSD when they were invited to participate, while others were introduced to the project as part of the initial assessment and planning of their treatment. Patients who expressed interest received additional information about the study from their treating psychologist, who also assessed eligibility and obtained informed consent. To be eligible, participants had to be aged 18 years or older, have a refugee background, receive a diagnosis of CPTSD based on the International Trauma Interview [19], be accepted for psychiatric treatment at CTTS, and be fluent in Danish to ensure meaningful participation in therapy and interviews without an interpreter.

### Intervention: Self-Tracking Assisted Psychotherapy

**Figure 2.**
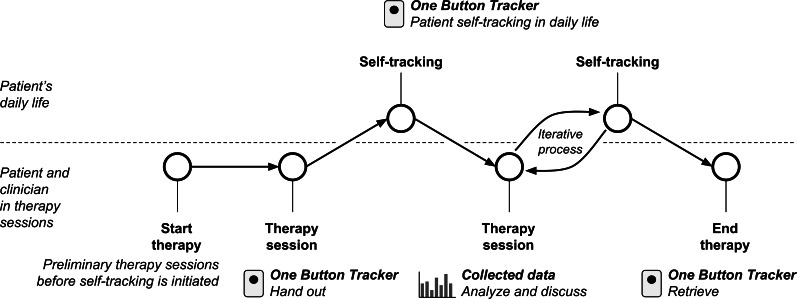
Flowchart outlining the self-tracking assisted psychotherapy process using the One Button Tracker (OBT).

The OBT was introduced to the patient by their treating therapist as a digital personal diary designed to track subjectively defined and relevant mental health phenomena during daily life [14,20]. This specific phenomenon is referred to as the target phenomenon, which is pertinent to the patient’s unique mental health challenges (Figure 2 for an outline of the treatment process). Together in the therapy session, the patient and therapist identified and defined the target phenomenon (eg, a symptom, impulse, or behavior) to be tracked. The target phenomenon should present itself clearly to the patient when it occurs; therefore, patients were encouraged to identify somatic or behavioral markers associated with its occurrence [14]. This target phenomenon could be adjusted throughout the treatment period based on the patient’s experiences with tracking and the insights gained from therapy sessions. Patients were encouraged to carry the OBT with them during their daily routines, using it as a wristband, around their neck, or in a pocket or bag.

Figure 2 displays the flowchart, which illustrates the collaborative definition of target phenomena, data collection in daily life, and therapeutic reflection using a web-based visualization tool. This process was developed and evaluated between 2022 and 2024 in a Danish clinical study with refugee patients diagnosed with complex posttraumatic stress disorder.

**Figure 3.**
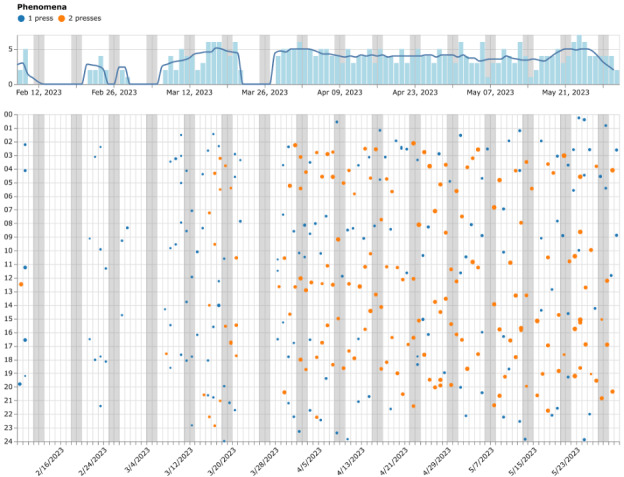
Calendar visualization of self-tracking data from participant P2 in a clinical qualitative study with refugees diagnosed with complex posttraumatic stress disorder.

Based on the patients’ expectations of the occurrences of the target phenomenon during their daily life, the therapist and patient developed a hypothesis list to predict patterns and dynamics in the target phenomenon. An observation protocol was then created to specify tracking criteria, including characteristics of the target phenomenon and how the OBT would be carried. Initially in therapy, patients could choose 1 phenomenon to track during their daily life. If deemed relevant, patients were introduced to the option of tracking 2 phenomena, assigning single or double button presses accordingly [20]. Frequently, a single press represented a distressing phenomenon, while a double press corresponded to having completed a therapeutic intervention at home as agreed upon in therapy. Upon returning to the therapy session, the collected data were analyzed collaboratively using a web-based data visualization tool as illustrated in Figure 3. This figure comprises blue dots representing single button presses (typically associated with distressing events), and orange dots representing double presses (often indicating completed interventions). Dot size reflects press duration. Data were collected using the One Button Tracker as part of an intervention conducted at a Danish trauma clinic between 2022 and 2024. These discussions focused on developing a shared understanding of the occurrences of the target phenomena and personalizing interventions based on the insights gained from the data.

### Interviews

The interviews were semistructured, meaning they followed an interview guide but remained flexible to allow exploration of new and unexpected insights into patients’ lived experiences [21]. The interview guide was based on both a clinical and a postphenomenological framework, focusing on patients’ interactions with the OBT across 3 key areas: its role in their treatment (eg, “What have you and your therapist decided your target phenomenon should be?”), their experiences of using the OBT in daily life (eg, “How has having the OBT with you in your daily life been?”), and its impact on their social relationships (eg, “Have you shown your family and friends the OBT?”). A total of 25 interviews were conducted with 9 patients at 3 stages: before, during, and after treatment. Two patients participated in only 2 interviews, due to the short duration of their treatment.

Participants were given the option to participate in the interview either at the clinic or in their own homes. Interviews were conducted face-to-face in Danish by the first author (LGR) except for 1 interview where LGR served as the therapist; in this case, author (SBM) conducted the interview. The duration of the interviews ranged from 19 to 93 minutes, with a median of 53 minutes. At the clinic, interviews were held in consultation rooms. In total, 22 (88%) interviews took place at the clinic, while the remaining 3 interviews were conducted in patients’ homes (2 patients). All interviews were audio-recorded and subsequently transcribed verbatim by a research assistant. To ensure accuracy, LGR cross-checked all transcripts against the audio recordings. Quotations presented in this study have been translated from Danish into English by LGR. Each quote is accompanied by a patient ID to ensure anonymity.

### Data Analysis

The analysis was conducted by LGR under the supervision of co-authors [JEL, TBC, JA, and LH] using reflexive thematic analysis as described by Braun and Clarke [22,23]. An inductive approach was used, grounded in a postphenomenological theoretical framework [17,18,24], which provided the lens for understanding how the OBT mediated patients’ experiences in their everyday life. In this analysis, we drew on Ihde’s [17] 4 human-technology relations to interpret patients’ engagement with the OBT: technologies can become embodied as extensions of the body (eg, glasses), function as hermeneutic relations representing reality (eg, a thermometer), be experienced as quasi-others in alterity relations (eg, chatbots), or recede into the background as part of the environment (eg, noise from a refrigerator). Importantly, these relations are not mutually exclusive. Instead, as Ihde [17] argues, technologies exhibit multistability, the ability to take on different roles depending on the user’s context and intentions [24,25]. According to Ihde [17], technologies are inherently ambiguous and transform the user’s experience as per the cultural context in which it is used.

The analysis followed Braun and Clarke’s [23] 6-phase reflexive thematic analysis, which emphasizes flexibility, researcher reflexivity, and the interpretive nature of thematic development. In the first phase, LGR familiarized herself with the dataset by listening to audio recordings and reviewing the interview transcripts multiple times. This immersion allowed for an in-depth understanding of both explicit content and latent meanings embedded in the patients’ narratives. In the second phase, initial codes were generated to identify significant features of the data relevant to the research question. Codes reflected variations in patients’ experiences with the OBT and were iteratively refined as patterns were identified.

The third phase involved clustering the codes into initial themes, reflecting patterns related to the OBT’s role in the lives of the patients. These themes were developed and reviewed iteratively throughout phases 3 and 4 through repeated engagement with the data and ongoing discussions with coauthors to ensure coherence and alignment with the study’s research question. The postphenomenological framework served as a theoretical lens during this phase, allowing us to interpret the data in terms of how the OBT mediated patients’ experiences through its multistability. In phases 5 and 6, themes were refined, clearly defined, and named to ensure that they captured the nuances of patients’ perspectives while using the theoretical framework as an analytical resource and contextualizing findings within existing literature.

### Ethical Considerations

This study was reviewed and approved by the Regional Committees on Health Research Ethics for Southern Denmark (project ID: S-20210019 CSF) and conducted following the Declaration of Helsinki. All participants received written and oral information about the study before participation and provided informed consent. This included consent for the use of anonymized quotations, interviews, and self-tracking data. No financial compensation was provided to participants. To protect privacy, all data were pseudonymized and securely stored following Danish data protection regulations. Interview transcripts and tracking data were deidentified, and access was restricted to members of the research team. No personally identifying information appears in the manuscript or supplementary materials, and all figures presenting self-tracking data have been fully anonymized to ensure confidentiality.

## Results

### Overview

In total, 9 patients participated in this study (n=6 females, n=3 males), aged 22-63 years, with a median age of 47 years (IQR 31–57 years). Patients had been living in Denmark for an average of 21.4 years. Three patients were originally from Bosnia, while the remaining patients were from Caucasus, Lebanon, Iran, Afghanistan, Turkey, and Syria (refer to Table 1 for additional demographic data).

**Table 1.** Demographic characteristics of 9 refugee patients diagnosed with complex posttraumatic stress disorder, enrolled in a clinical qualitative study on the use of a novel wearable self-tracking instrument, the One Button Tracker, during psychotherapy.

Patient ID	Age (years)	Sex	Employment status	Years in Denmark
P1	31	Female	On sick leave	20
P2	63	Male	On sick leave	30
P3	59	Female	On sick leave	25
P4	22	Female	Student	22
P5	32	Female	Student	20
P6	57	Male	On sick leave	30
P7	52	Female	Temporary employed	15
P8	47	Male	Employed	22
P9	22	Female	Student	9

Table 1 presents the data collected at a specialized trauma clinic in Denmark between 2022 and 2024.

Patients used the OBT for durations ranging from 22 to 366 days and tracked between 2 and 14 different phenomena each throughout their treatment. Over this period, the number of recorded observations varied widely, from 37 to 4733, reflecting diverse patterns of engagement with the OBT (Table 2). The table includes the number of tracking sessions, duration of use, number of phenomena tracked, and button press frequency. Data were collected between 2022 and 2024 as part of a qualitative intervention study at a Danish trauma clinic.

**Table 2.** Individual-level summary of self-tracking activity using the One Button Tracker for 9 refugee patients with complex posttraumatic stress disorder.

Patient ID	Total sessions with tracking	Total days with tracking	Days with data	Days without data	Number of observations	Daily average observations	Protocols used	Target phenomena tracked
P1	12	92	62	30	1276	20.6	4	3
P2	17	112	87	25	347	4	4	4
P3	24	324	289	35	2038	7.1	17	14
P4	22	262	185	77	130	0.7	10	8
P5	13	366	278	88	980	3.5	8	8
P6	12	246	128	118	4733	37	5	5
P7	20	252	212	40	708	3.3	5	4
P8	8	175	58	117	188	3.2	1	1
P9	4	22	22	0	37	1.7	3	3

### Themes

The analysis resulted in five key themes: (1) from external instrument to extension of the self, (2) mental switch, (3) faithful companion, (4) scarlet letter, and (5) emergency lifeline. In the results that follow, we see how the OBT is multistable, functioning as an embodied extension of therapeutic interventions, a friend, a link to their therapist and treatment, and a scarlet letter.

#### From External Instrument to Extension of the Self

Patients varied in how they carried the OBT, reflecting individual preferences and comfort levels. In total, 5 opted for discrete placement, such as in a pocket, a bag, or a designated place in their home, while 4 patients wore it more openly on their wrist or around their neck. While these choices often remained consistent throughout treatment, a few patients adjusted their approach based on practicality or personal needs in specific contexts.

As the OBT became embedded in the daily routine of the patients, it evolved from being an external instrument to an extension of themselves. Within approximately 2 weeks, many patients described using the OBT as a natural and almost automatic part of their lives. One patient articulated this experience vividly:

*Yes. It’s like my body. Sometimes if I forget it, like if I’m driving, for example, to go shopping, then I go back, grab it and go again.*

[P7]

This embodiment was particularly evident in patients’ nighttime routines, where the OBT remained within reach. Some patients placed the instrument under their pillow, enabling them to record observations easily during the night. One patient shared how this practice became a habitual and embodied part of her routine:

*Well, at first I tried sleeping while wearing it, but in the middle of the night I noticed that I was very sore around my chest. So in the end, I just thought I’d put it under the pillow, so it’s easier for me.*

[P1]

*That makes sense.*

[Interviewer]

*Yes, it’s better. But sometimes when I wake up, without noticing, because I'm holding it.*

[P1]

*You hold it in the morning?*

[Interviewer]

*Without knowing why I've done it. So I hold it so much that I think I have it.*

[P1]

Why did the OBT become so indispensable to patients? For many, its significance extended beyond mere habitual use; it took on emotional and even physiological importance, integrating into their daily lives as an extension of their own bodies. This emotional attachment was especially pronounced when patients experienced the absence of the OBT, whether due to technical errors or returning the OBT at the end of treatment. The void left by the OBT was deeply felt, as 1 patient described a sense of loss when asked to stop using it:

*There was always something missing… I look at the spot where it used to be, you know? And then I say, “It’s not here.” Also, when I go out, you know? I miss it. I miss it a bit.*

[P6]

The physiological bond with the OBT was further reinforced by the tactile sensation of pressing the button, which patients came to associate closely with their emotional and physiological states. One patient vividly elaborated on the vibrotactile feedback’s profound effect, saying:

*It goes to my heart when I press. A lot. I feel it. It goes through the blood. It brings oxygen.*

[P3]

This enduring sense of presence, even when the OBT was no longer physically available, illustrates the OBT’s role as a stable extension of self-regulation that, once embodied, remains psychologically significant.

#### Mental Switch

For many patients, the OBT acted as more than a self-tracking instrument; it became a tangible instrument for self-regulation strategies, functioning as a mental switch that fostered a sense of agency during moments of distress. This role as a mental switch was characterized by multistability, adapting to the diverse intentions and emotional states of the patient. One patient illustrated how the act of pressing the button held different meanings depending on her intention of using it, while consistently carrying personal meaning:

*Or maybe it’s because, when I press the button, it starts to mean something.*

[P3]

*It means something?*

[Interviewer]

*Yes. It means maybe I’m anxious, maybe I’m scared, maybe I’m depressed, maybe it’s about my medication, or maybe I’m calling too often. It’s different each time. It’s starting to feel meaningful to me every time I press.*

[P3]

For some, this act of pressing the button was not merely functional but became a moment of reflective self-awareness, helping patients recognize and manage specific emotions or behaviors. The dynamic adaptability of the OBT allowed it to align with the unique needs of each patient, making it a personalized and integrated aspect of their therapeutic process. One patient described how the OBT became instrumental in resisting emotional eating, transforming an overwhelming impulse into a manageable act of self-regulation:

*It has helped a lot, really. Before, I knew I was eating away my feelings. But with pressing the button, you realize, “Okay, I have some feelings that I’m trying to eat away.” So, in a way, it stops it. Yes, it does something to the brain. Suddenly the brain is like, “Okay, I have some feelings, now I feel hungry, I should press, but I’ve pressed many times today. So I’ve eaten enough.” It gives something in the mind that suddenly makes you stop.*

[P9]

In these cases, pressing the button became a hermeneutic act, enabling patients to interpret their patterns and reflect on their impulses. This reflective process not only helped them resist impulsive behaviors but also fostered a sense of agency. At other times, the OBT receded into a background relation in the patient’s daily life, offering a constant and supportive presence.

For some patients, the act of pressing the button carried symbolic meaning, fostering hope and reinforcing their commitment to recovery. One patient described how the availability of the OBT as a mental switch gave her reassurance in moments of anxiety:

*Yes, if it isn’t there… then it’s like I’m empty, like I’m missing someone. When anxiety comes, what should I do? Yes, of course, we have many strategies. But it’s also… I don’t know. But if I press the button, I believe I will get better.*

[P7]

For others, pressing the button offered emotional regulation during moments of distress, helping them regain a sense of agency. One patient shared how the OBT helped him manage intrusive and distressing thoughts:

*When I think about the war. When I think about my village. When I think about everything. Then I press too. It’s like... How should I explain it? It’s like it says to me: “Relax, it will be okay.”*

[P6]

Here, the act of pressing the button was shaped by the therapeutic context in which the OBT was introduced in, its macroperception, which informed how the sensory experience of pressing the button, or microperception [18], was interpreted. This context imbued the act with a sense of agency, enabling patients to interrupt intrusive and distressing thoughts. For 1 patient, this process symbolized a mental switch, allowing her to shift distressing thoughts and reinforcing her connection to therapy:

*When I press the button, it shifts my bad thoughts so quickly. But it comes back again. But I have to do it because it helps me. That’s because, for example, when you don’t use the light here, right? When you go outside, you turn off the light, right? It helps me so much.*

[P5]

Over time, the OBT became an anchor that paused impulsive behavior and allowed time for thoughtful decision-making. Patients noted how pressing the button encouraged them to slow down and reconsider their actions. One patient articulated this shift:

*But I would say that the button itself has, in a way… I don’t know, somehow made me think twice about things and not just do things impulsively. It’s actually given me a chance to be a bit more sensible in some situations and really think things through.*

[P4]

For many patients, pressing the button became a meaningful act, encouraging emotional regulation and self-reflection during moments of distress. Reflecting on her use of the OBT, 1 patient described how it helped her reconnect with her emotions:

*Well, it has somehow forced me to feel something. Or at least to check in with myself and feel something. I’ve been pretty numb in relation to my emotions. But over time, it’s gotten me to feel more. And it’s strange, since it’s just a button, but it’s gotten me to feel more. Because I’ve been forced to do it. I’ve also forced myself to do it. And I don’t even know, I don’t think I ever really understood its purpose. I knew it was supposed to track a lot of things, but I didn’t know exactly for what purpose. But for me, the purpose has been completely personal, that it could get me to feel again.*

[P4]

This theme reflects the depth and complexity of the act of pressing the button of the OBT. While appearing as a simple and insignificant act, it served as a mental switch, a way for patients to regulate their emotions, provide hope for recovery, and intentional self-awareness in the moments they needed it the most.

#### Faithful Companion

For 7 patients, the OBT transcended its role as a self-tracking instrument, assuming the role of a “quasi-other” that provided support. Patients consistently anthropomorphized the OBT, attributing it with a sense of agency and giving it a “voice” that offered them encouragement and companionship. For example, 1 patient, described feeling like the OBT was an advisor, guiding her decisions and reinforcing behavior change:

*It’s like it says to me: “[Her name], you have a lot of anxiety. [Her name], why don’t you go outside? [Her name], why aren’t you sitting with others and talking like everyone else?”*

[P3]

This sense of connection and guidance was closely tied to the OBT’s vibrotactile feedback, which, for some patients, became an affirmation of support that symbolized companionship in moments of distress. The vibrotactile feedback, in particular, was perceived as a sign of life, enhancing the felt connection between the patient and the instrument. One patient articulated this as a feeling of not being alone:

*It was like there was someone with you the whole way. Yes. You get this feeling that you’re not alone in this process. There’s someone who is also keeping an eye on what you’re doing. Or on your challenges. Feeling the vibration was just wonderful. It was just great. Because it’s like it responds to you.*

[P9]

Here, the tactile feedback served not just as a technical acknowledgment of a recorded observation but mediated an emotional relation, making the OBT feel like a quasi-other that provided comfort during challenging moments and in periods of solitude. One patient described how he held the OBT in his hands at night as if it were a supportive friend, substituting for his wife’s presence:

*How should I say it? Because the others are sleeping. And I mostly wake up at night. I wake up many times. So I take it in my hand and hold it. Instead of holding my wife, for example. She’s sleeping; she has to work in the morning, so I hold it. It’s like it helps me. “I’m here,” for example. In that way.*

[P6]

For this patient, the alterity relation was disrupted during technical outages, which revealed the extent of their emotional reliance on the OBT. When the vibrotactile feedback stopped functioning, the patient expressed it as a loss akin to losing a friend:

*It’s not the same. It’s like my friend has died. Because he doesn’t respond. So if it vibrates, then it’s like, “There’s life in you.” Then it’s fine.*

[P6]

In these moments, the OBT’s quasi-other role in patients’ lives became evident, embodying a form of companionship that fostered a therapeutic extension beyond the clinical setting.

#### Scarlet Letter

The OBT served as a visible, purpose-specific object in patients’ daily lives, shaping their self-awareness and social interactions. While its physical presence bridged the gap between therapy and everyday life, it also introduced challenges and opportunities, amplifying or reducing engagement depending on the context.

For 3 patients, the visibility of the OBT took on the meaning of a scarlet letter of shame leading to discomfort and self-consciousness, particularly in public or familial settings. One patient explained how its presence conflicted with her preference for keeping her mental health challenges private:

*Well, I don’t need people to see it. Like, starting to ask questions, and I don’t need people to know that side of me. My colleagues know that I see a psychologist, but they don’t need to know more than that. So, I’m fine with not wearing it. Because then it becomes obvious that something’s going on.*

[P4]

Another patient described how the visibility of the OBT influenced family dynamics, amplifying family members’ negative views toward mental health care treatment. One patient recounted how his teenage daughter disliked seeing him wearing the OBT, as she expressed how she feared it might lead to him being taken away and institutionalized. This patient also shared how his wife discouraged him from wearing the instrument around extended family, reflecting concerns about social stigma:

*She [his wife] doesn’t want me to bring it with me. She says we should stay together, and you don’t need to have it with you. Maybe she... Because we have family down there, and maybe they would ask, “What is that?” All sorts of things. So maybe she’s ashamed or something. That could be.*

[P6]

Although this patient was comfortable wearing the OBT among family members, his wife’s discomfort led him to stop using it in certain settings. The visibility of the OBT amplified existing stigmas surrounding mental health treatment, which ultimately influenced his engagement with the instrument.

Conversely, for some patients, the OBT’s visibility transformed from a scarlet letter of shame into a symbol of strength and proactive management of mental health. One patient shared how her daughter supported her use of the instrument:

*I sometimes forget it [the OBT] when I’m lying down somewhere. My daughter says, “Mom, I brought your instrument”, and then she places it under the pillow for me. She reminds me of it.*

[P1]

In this instance, the OBT encouraged familial support and established a sense of shared responsibility in the therapeutic process. This dynamic not only enhanced the patient’s accountability but also strengthened family bonds, contrasting the negative associations of the scarlet letter with a sense of empowerment. While some patients found the OBT supportive, others struggled with its aesthetics, which created mixed feelings about wearing it openly. One patient remarked on its appearance:

While some patients found the OBT supportive, others struggled with its aesthetics, which created mixed feelings about wearing it openly. One patient remarked on its appearance:

I wasn’t happy with it. Like, because it was ugly and looked like an ankle monitor. It was kind of a bit of a love-hate relationship we had. Because it was so ugly, but it still made me think about what I was tracking.

[P4]

For her, the OBT’s appearance compromised her willingness to wear it, evoking associations with social judgment similar to the stigma of a scarlet letter. Similarly, another patient found that the OBT’s appearance triggered memories of wartime imprisonment:

And I haven’t received much information. What does it mean for me? At first, I thought: “Oh, for hell’s sake, it’s like I’m back in prison.” Like I have to report to the police that I’m here.

[P2]

These varied reactions illustrate the OBT’s multistability, highlighting its ability to take on different roles and meanings depending on the specific contexts and situations in which it is used. For some contexts, the OBT served as a source of stigma and discomfort, while in others, it fostered engagement and support, underlining how the meaning of the instrument is not fixed, but rather shaped by the circumstances in which it is integrated.

#### Emergency Lifeline

The OBT served as an emergency lifeline for many patients, facilitating nonverbal communication that bridged their daily lives and therapy sessions. The OBT mediated patients’ inner dialogues, reinforcing the therapeutic relationship and acting as an embodied extension of emotional communication. One patient described how pressing the button created a sense of being “seen” by her therapist, even outside of sessions:

*It was kind of like: “I’m eating my feelings away. Please help me.”*

[P9]

*And what happens when you press, and you sort of say, please help me? What happens then?*

[Interviewer]

*Yes, well, then I can feel that, okay, I’m not alone in this. My therapist will see it, maybe she’ll do something about it or say something to me that helps. Figure out why I’ve eaten so much this past week, for example.*

[P9]

This interaction established a feedback loop, strengthening the therapeutic relationship and fostering a sense of accountability. Another patient highlighted how pressing the button encouraged reflection on therapy sessions:

*I feel like I'm sitting right in front of my therapist, and we're talking. What did we talk about last time? What helps me?*

[P5]

In such moments, pressing the button allowed patients to “call on” their therapist’s advice, creating a silent connection to their treatment. Similarly, another patient described how pressing the button created a sense of support in moments of need:

*It’s like someone is saying to you: “I’m here.”*

[P9]

*Yes. So, when you press it and it vibrates?*

[Interviewer]

*Yes. It’s like my whole body felt that someone is here. Right here beside me. But it also made me feel like something in my mind, like my therapist is with me all the time because of the tracker.*

[P9]

In such moments, the OBT mediated an immediate and nonverbal imagined dialogue with the therapist, fostering a sense of support during the patient’s daily life. In both cases, the OBT mediates a nonverbal dialogue with the therapist, allowing patients to feel supported between sessions. As such, the OBT embodied the therapeutic relationship, reinforcing patients’ sense of accountability and engagement. One patient described how the OBT served as a daily reminder of her therapeutic homework:

*As I told my therapist, it’s kind of like a task for me, actually. That’s how I see it. It’s a task for me, so it’s supposed to help me. So I don’t know when it will help me, but that’s how I think about it.*

[P2]

The OBT thus channels patients toward therapeutic engagement, embodying both the presence and expectations of their therapist in their everyday lives. For some, even after returning the OBT, its influence persisted, though not as strongly.

## Discussion

### Principal Findings

This study is the first to explore how refugees with CPTSD engage with a single-purpose, wearable (the OBT) to track a personally relevant and collaboratively defined mental health phenomenon in daily life. The findings showed that the OBT-mediated therapy promoted agency, helping patients regulate emotions and stay connected to their therapist between sessions. As an active mediator, it shaped how patients related to their symptoms, treatment, and themselves. These findings reveal both the potential and limitations of the OBT, offering a more nuanced understanding of how self-tracking can support psychotherapy.

### Design Simplicity and Integration

The OBT’s minimalistic design made it easy to incorporate into daily life. Most patients described it as intuitive within the first 2 weeks, aligning with previous research showing that low cognitive demand improves engagement [2,26]. Its simplicity allowed patients to register experiences in the moment without distraction, creating a sense of immediacy that supported embodied engagement. Some even described the instrument as becoming part of their body.

These experiences can be understood through Ihde’s [17] concept of multistability and Verbeek’s [18] theory of technological mediation, which describe how technologies shape human intentionality and experience. As the OBT faded into the background, patients could focus less on the instrument itself and more on the intention of pressing the button. In this way, the OBT became an active part of the therapeutic process, not just an instrument for collecting data, but something that supported agency and intentionality in the moment.

At the same time, the simplicity of the design of the OBT placed responsibility on the patient. With no structured prompts or categories, patients had to remember their target phenomena and interpret what their data meant over time. Several adapted or expanded their target phenomena (from one to two target phenomena) as therapy progressed. While this flexibility enriched the therapeutic process, it also required therapists to stay attuned to how patients were using the OBT and what their patterns meant. Supporting this evolving engagement calls for an open and adaptive therapeutic approach.

Taken together, these findings suggest that simplicity and personalization are central to making self-tracking meaningful in a therapeutic context. When patients define what is relevant to track, the OBT can support a stronger sense of agency and bring therapy into the flow of everyday life. This reframes the role of wearable technologies in mental health, not just as passive data collectors, but as instruments that actively mediate change. At the same time, striking the right balance between openness and support is key. Simplicity must not come at the cost of clarity or overwhelm patients with too much interpretive responsibility.

### Personalization Through Multistability

The OBT’s multistability [17] was central to how patients engaged with it. Although pressing the button was a uniform act, the meaning and function varied widely. Some patients described it as a “mental switch” that helped them pause or reflect in difficult moments. Others experienced it as a “faithful companion” that offered emotional support between sessions. A few used it as a silent link to their therapist, an “emergency lifeline” that created a sense of connection and accountability.

One patient described how the vibration moved through her body “like blood,” illustrating how the OBT, in some cases, became more than a tool. It was experienced as part of the self. This points to what Verbeek [25] describes as a cyborg relation, where the boundary between person and technology dissolves. Here, the OBT no longer simply mediates therapeutic engagement, but shapes how patients experience themselves and their treatment.

This deep integration may support agency, but it also raises concerns about dependency. Two patients asked to keep or buy the OBT after treatment, and 1 delayed their final session to continue using it. While strong engagement can be beneficial during therapy, it may complicate the end of treatment. Therapists using similar tools should be prepared to help patients gradually internalize what the instrument supported, so that progress continues without it.

The vibrotactile itself played an important role. For many, it was not just feedback, but a form of affirmation. The immediate tactile response gave a sense of being acknowledged, reinforcing their actions and sometimes symbolizing the presence of the therapist. Unlike tools that provide delayed or abstract feedback [27], the OBT offered something more grounded in both the body and the therapeutic relationship.

These findings highlight the value of personalization, especially when contrasted with existing literature that has primarily focused on collecting physiological, contextual, or environmental data to tailor ecological momentary interventions [9]. While such approaches often emphasize objective metrics, the OBT allowed patients to define for themselves what was relevant to track in daily life. This made the act of self-tracking more personally meaningful and emotionally resonant. It also provided detailed, in-the-moment collected data that therapists could draw on to guide conversations and adapt treatment in ways that felt more aligned with each patient’s lived experience.

Still, the multistability of the OBT sometimes introduced challenges. Not all of the patients’ interpretations were helpful. One patient believed the vibration would “absorb” his anger and felt disappointed when that did not happen. These examples show why clear communication is essential when introducing wearable instruments. Patients need guidance and support to make sense of what the OBT can and cannot do, and how to use it in ways that strengthen rather than disrupt the therapeutic process [28].

### Visibility and Its Dual Impact

The visibility of the OBT mediated how patients managed their self-presentation in everyday life. While the instrument often blended into daily routines [17], it also acted as both a supportive and disruptive mediator. For some, it encouraged openness and relational support; for others, it amplified feelings of exposure and stigma. Drawing on Feenberg’s [29] expansion of Ihde’s [30] concept of the extended body, the OBT served both as a medium of self-awareness and as a visual representation of the patient. This dual role reflects the complexity of visible self-tracking technologies. The OBT’s physical presence influences how patients navigated social and cultural expectations around mental health. While some welcomed the visibility as a sign of taking action, others found it uncomfortable, comparing it with an ankle monitor.

These findings raise broader questions about the design of wearable health technologies. As Nunes et al [27] argue, devices that look overly clinical may alienate users, particularly in contexts where mental health remains stigmatized. While mobile technologies are often preferred for their neutral appearance [9,31], they also face high dropout rates and low engagement [32]. The OBT’s single-purpose design appears to support stronger therapeutic involvement, but its visibility may also create barriers in settings where discretion is essential.

This suggests that therapists should actively explore the social contexts in which patients will use such wearable technologies. Helping patients prepare for how the OBT may be interpreted by others can reduce discomfort and support sustained use. Future research should also consider how these dynamics unfold across different cultural settings, where visibility and stigma may carry distinct meanings.

### Limitations

This study has some limitations. First, none of the participants were native Danish speakers, which may have affected the depth and nuance of their interview responses. Language barriers likely limited their ability to fully articulate their experiences with the OBT. The decision to exclude interpreters and avoid translation was a deliberate trade-off, made to prioritize the authenticity of the patients’ voices. However, this approach also meant that patients had to express themselves in Danish, which could lead to some loss of depth and nuance in their responses. In addition, limiting the sample to Danish-speaking patients may have excluded newly arrived refugees who had not yet learned the language, thereby limiting the cultural diversity of the sample and reducing visibility of culturally specific experiences with the OBT. Future studies could address this limitation by incorporating trained interpreters who are familiar with both the language and the study context, ensuring accurate and reliable data collection from nonnative speakers.

Second, the dual role of LGR as both researcher and PhD candidate may have influenced participants’ responses, as evidenced when 2 participants inquired about LGR’s academic progress. This interaction suggests participants’ awareness of the researcher’s personal stake in the study, potentially biasing their accounts toward more favorable descriptions of the OBT. To overcome this, interview questions were carefully designed to explore both positive experiences and challenges related to OBT use, ensuring a balanced and comprehensive perspective.

### Conclusion

This study explored how the OBT mediates patients’ psychotherapeutic process in daily life. Addressing the research question, our results show that the OBT actively shaped patients’ engagement with therapy by fostering agency, emotional regulation, and a sense of connection to their therapeutic process beyond clinical sessions.

The OBT was integrated into patients’ daily routines, where it took on multiple roles based on their needs and contexts. These results challenge the traditional emphasis on passive data collection in mental health technologies, showing that self-tracking instruments can act as active mediators of therapeutic processes. Through its vibrotactile feedback and the act of tracking, the OBT became a tangible tool for self-awareness, nonverbal communication, and agency in moments of need. This study emphasizes the need for digital mental health technologies to focus not only on data but on their relational and experiential dimensions. Future research should build on these findings, examining how wearable technologies mediate therapeutic processes across diverse contexts and exploring design improvements to address issues such as stigma and visibility.

Taken together, the OBT illustrates how a simple, single-purpose, wearable self-tracking instrument can mediate patients’ treatment during their daily lives, thereby bridging the gap between therapy sessions and real-world challenges. These insights contribute to a deeper understanding of how design principles for personalized wearable self-tracking instruments can support mental health care beyond passive data collection.

## Abbreviations

CPTSD: complex posttraumatic stress disorder
CTTS: Clinic for Trauma and Torture Survivors
OBT: One Button Tracker
